# The Relationship between Betatrophin Levels in Blood and T2DM: A Systematic Review and Meta-Analysis

**DOI:** 10.1155/2016/9391837

**Published:** 2016-05-03

**Authors:** Song Yue, Jingyang Wu, Jiahua Zhang, Lei Liu, Lei Chen

**Affiliations:** ^1^Department of Ophthalmology, The First Affiliated Hospital of China Medical University, Shenyang 110001, China; ^2^Department of Epidemiology, School of Public Health, China Medical University, Shenyang 110001, China

## Abstract

*Background*. In order to clarify previous ambiguous research conclusions, a meta-analysis was made to investigate the relationship between betatrophin levels in blood and type 2 diabetes mellitus (T2DM).* Methods*. We have searched all the English and Chinese references regarding the relationship between betatrophin and diabetes in database both manually and online. Strict criteria have been established to include and exclude articles, with Mean and Standard Deviation as statistics to evaluate strength of association. We have chosen either fixed- or random effect model according to heterogeneity inspection results and used Begg's test and Egger's test to analyze publication bias.* Results*. A total of 11 studies were included in this meta-analysis. Meta-analysis indicated a significant association between betatrophin and T2DM (Mean: 329.46; 95% confidence interval: 182.51 to 476.42, *P* < 0.0001). However, in the subgroup analysis, there was no significant statistic between betatrophin concentration and T2DM within Caucasian population (Mean: 98.40; 95% confidence interval: −1585.08 to 1781.88, *P* = 0.91).* Conclusions*. Such relationship may suggest preference for association between betatrophin and T2DM in different population.

## 1. Introduction

Diabetes mellitus (DM) is a type of metabolic disease characterized by hyperglycemia. It is caused by either defected insulin secretion or damaged biological function, or both. The statement of a high-level blood glucose that a body is in for a long time will lead to dysfunction of a variety of tissues. Nowadays, such methods as taking antidiabetic medicines or injecting insulin to cope with diabetes are the usual practice, but there is no way of thorough treatment. Recently, researchers [1] from Harvard Stem Cell Institute (HSCI) found a new hormone and named it betatrophin, which, closely related to diabetes treatment, could increase the quantity of cells that produce insulin in mice quickly. Betatrophin, mainly circulated in liver and adipose tissues, promotes greatly the proliferation of pancreatic beta cells, plays an important role in modulating glycolipid metabolism, and maybe replaces insulin in the effective treatment of diabetes. Before that, different research teams name it differently according to its different functions, including angiotensin amyloid 8 (ANGPTL8) [2], lipoprotein lipase inhibition (Lipasin) [3], and refeeding induced in fat and liver (RIFL) [4]. Previous reports [5] showed that serum betatrophin levels were significantly higher in T2DM, while others showed that the expression of betatrophin index declined [6], and the third conclusion was that there was no statistical significance on the relationship between betatrophin levels and T2DM [7]. In order to evaluate the correlation between betatrophin and diabetes objectively, we perform a meta-analysis on related studies that are qualified, in the hope of getting a more persuasive conclusion.

## 2. Methods

### 2.1. Selection of Eligible Studies

Two researchers (Yue and Wu) have conducted document retrieval to the relationship between betatrophin and DM independently and elaborately, who have searched such database in the text as PubMed, MEDLINE, Embase, Cochrane Library with “betatrophin and diabetes,” “Angptl8 and diabetes,” “RIFL and diabetes,” and “Lipasin and diabetes” combined and searched others including CNKI, China Wan-Fang database, and Chongqing VIP database with “betatrophin and diabetes.” The latest search date is May 1, 2015. We have studied every article selected as “Related Articles” by PubMed and searched it to get extra potentially related articles. We have also searched the references and contacted the authors to get extra articles. When there was ambiguity about the results or lack of sufficient data, we contacted all authors to make it clear. The searching method has been made beyond linguistic limits, but we only included the articles published in English or Chinese language.

### 2.2. Selection Criteria

The following criteria have to be met for the articles to be included: (1) case-control study; (2) the cases in those studies that were type 2 diabetes; (3) all the case groups which were in accordance with international criteria for diagnosis of type 2 diabetes; (4) articles published as papers until May 2015; (5) articles that provided directly or indirectly the results on relevant research index in case group and control group.

### 2.3. Exclusion Criteria

Exclusion criteria are defined as follows: (1) articles in which the case group was diagnosed with type 1 diabetes; (2) research with insufficient data; (3) research with data that could not be converted; (4) adopting the best-quality among research papers that were duplicated, repeatedly collected, or with similar data.

### 2.4. Data Extraction

The two researchers entered into each database, respectively, with standard procedures and extracted research data independently. If there was a discrepancy, the researchers would appraise the data together. The following information was gathered including the first author, publication year, nationality, blood sample, experimental method, sample size of patients and the control group, and Mean and Standard Deviation (SD) (part of the data were converted) of betatrophin levels.

The review and analysis were guided to conduct by the PRISMA statement for preferred reporting of meta-analysis [16].

### 2.5. Statistical Analysis

The RevMan5.3 statistical software provided by The Cochrane Collaboration was applied, with Mean and SD as statistics to evaluate strength of association. According to heterogeneity inspection results, corresponding pooled method was chosen: when there was no significant heterogeneity in research results (*I* ^2^ < 50%), the fixed effect model was used to weight and pool the effect size. However, when research results appeared to be heterogeneous (*I* ^2^ > 50%), the random effect model was adapted to weight and pool the effect size, and meta-analysis forest plot was drawn. We used statistical software named Comprehensive Meta-Analysis (http://www.meta-analysis.com/pages/demo.php) to draw funnel plots, and an asymmetrical one suggested the existence of heterogeneity. Besides, the researchers used Begg's and Egger's test to analyze publication bias, and it is considered that if *P* < 0.05 in Begg's and Egger's test, publication bias was significant statistically. All *P* values were two sided.

## 3. Results

### 3.1. Study Inclusion and Characteristics

**Figure 1:**
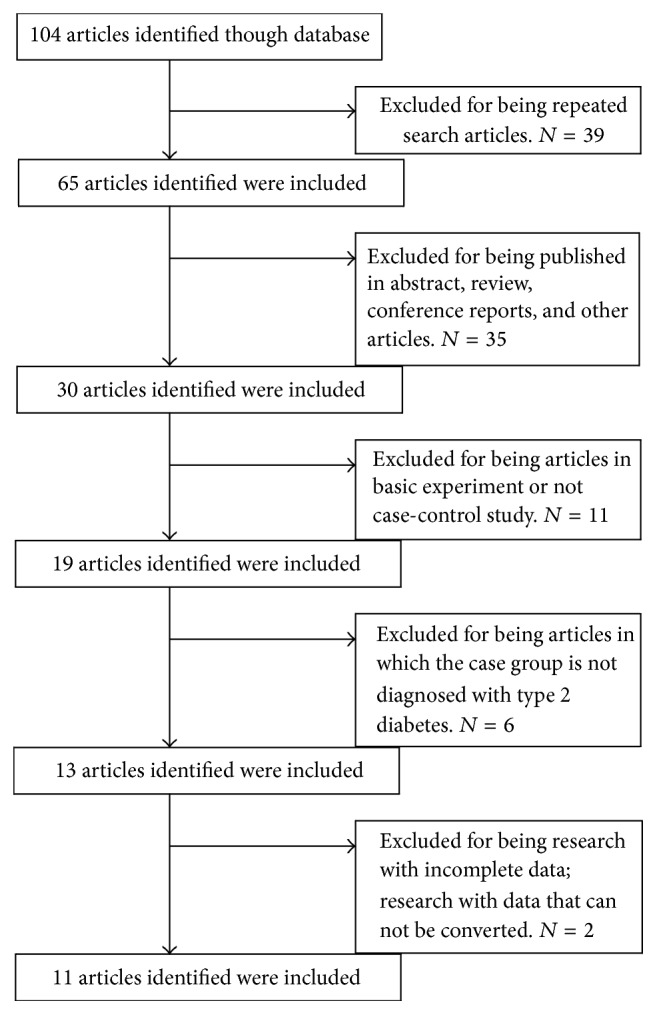
Flowchart demonstrating those studies that were processed for inclusion in the meta-analysis.

After the initial screening, there were 104 papers which reached screening stage including 71 in English and 33 in Chinese. After strict selection process, a total of 11 articles [5–15] remained for this meta-analysis. The results of the selection process were displayed in Figure 1. There were nine English literatures and two Chinese publications with 490 cases and 508 controls. The test samples were plasma in two literatures and serum in the rest nine, and all the test methods in included literatures were enzyme-linked immunosorbent assay (ELISA) method. The characteristics and findings of these experiments were presented in Table 1.

### 3.2. Outcomes

**Figure 2:**
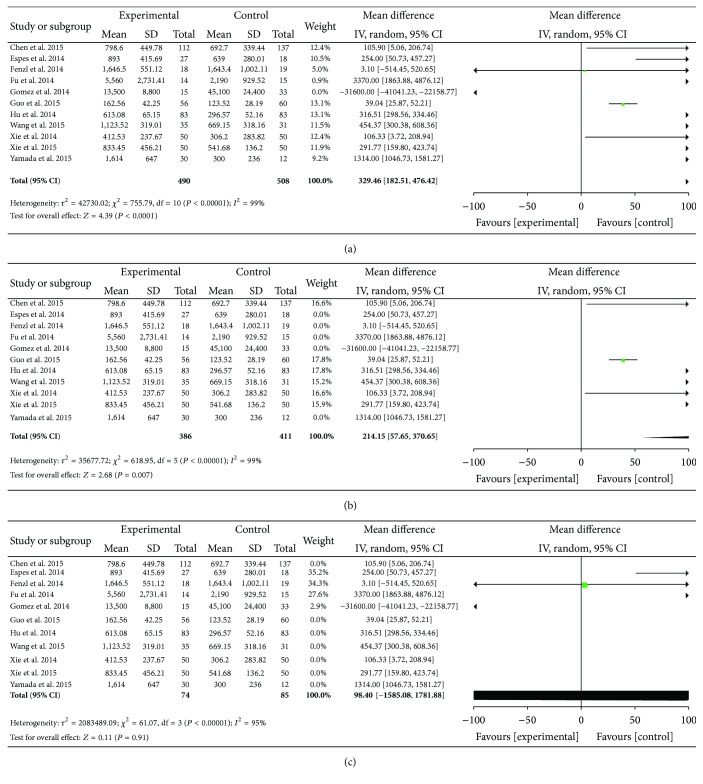
(a) Forest plot of the circulating level of betatrophin in T2DM patient, studies are pooled with random-effects model. (b) Forest plot of the circulating level of betatrophin in Chinese T2DM patient, studies are pooled with random-effects model. (c) Forest plot of the circulating level of betatrophin in Caucasians T2DM patient, studies are pooled with random-effects model.

**Figure 3:**
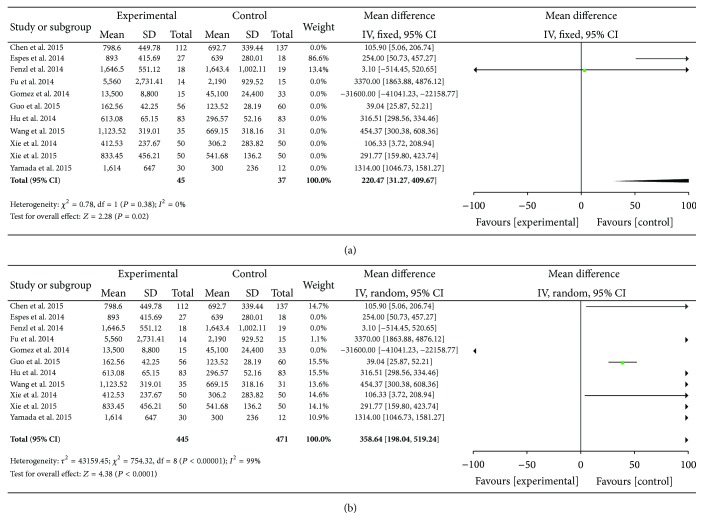
(a) Forest plot of the circulating level of betatrophin in T2DM patient plasma. (b) Forest plot of the circulating level of betatrophin in T2DM patient serum.

All this meta-analysis outcomes were shown in Figures 2 and 3. (1) It is obvious that heterogeneity existed in all the eleven included papers (*P* < 0.00001, *I* ^2^ = 99%). Therefore, the random effect model was employed to conduct pooled analysis. The outcomes of pooled Mean [95%  CI] at 329.46 [182.51,476.42], *Z* = 4.39, showed that the circulating level of betatrophin in T2DM patient blood was higher than that in control groups, in Figure 2(a).

(2) A subgroup analysis was carried out based on different group of people. The results showed that heterogeneity (*P* < 0.00001, *I* ^2^ = 99%) existed in the six papers that performed the study on Chinese people and therefore the random effect model was used to do meta-analysis. The results of pooled Mean [95%  CI] at 214.15 [57.65,370.65], *Z* = 2.68, revealed in Figure 2(b) that the circulating level of betatrophin in Chinese T2DM patient blood was higher than that in control groups. Likewise, heterogeneity (*P* < 0.00001, *I* ^2^ = 95%) was more significant in the four papers that studied Western Caucasians and therefore the random effect model was used to do pooled analysis. As the result, pooled Mean [95%  CI] was at 98.40 [−1585.08,1781.88], *Z* = 0.11, which showed that the index of betatrophin circulating level in Caucasians' blood had no statistical significance, in Figure 2(c).

(3) Another subgroup analysis was made based on different types of blood samples. The two papers with plasma as research sample did not present a conclusion of heterogeneity (*P* = 0.38, *I* ^2^ = 0), and the fixed effect model was chosen to do pooled analysis. The results of pooled Mean (95%  CI) at 220.47 [31.27,409.67], *Z* = 2.28, revealed in Figure 3(a) that the plasma concentrations of betatrophin are higher in T2DM patient.

In addition, the random effect model was carried out to do pooled analysis according to the result of heterogeneity (*P* < 0.00001, *I* ^2^ = 99%) in the nine papers with serum as research sample. The results of pooled Mean (95%  CI) at 358.64 [198.04,519.24], *Z* = 4.38, revealed in Figure 3(b) that the serum concentrations of betatrophin are also higher in T2DM patient. All the forest plot data summary was in Table 2.

**Figure 4:**
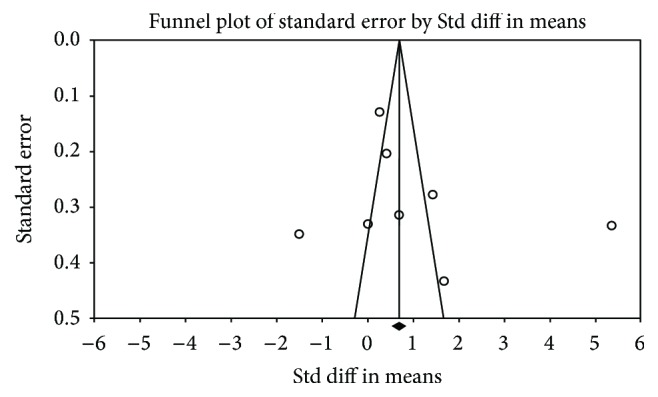
Funnel plot based on 11 case-control studies.

According to Begg's and Egger's tests (Begg, *P* = 0.11; Egger, *P* = 0.25), the funnel plot was not asymmetrical (Figure 4), which demonstrated a nonsignificant *P* value publication bias.

## 4. Discussion

DM is a metabolic disorder caused by pancreatic beta cells defect or damage [17], characterized by increased chronic hyperglycemia level. Long-term hyperglycemia can cause damage to multiple systems [18]. To date, main therapeutic approach in treating diabetes is to improve insulin resistance, promote insulin secretion, or preserve the remaining beta cell function by using insulin or drugs [19]. Although it can control the blood glucose to some extent, it cannot solve the fundamental problem: relative or absolute pancreatic beta cells deficiency.

Previous report showed that betatrophin-encoded protein could significantly promote the proliferation of mouse pancreatic beta cells with increasing number so as to enhance glucose tolerance. With these striking study findings, many scholars conducted research on this newly discovered peptide and examined the relevance between betatrophin and T2DM by detecting the circulating levels of betatrophin in T2DM patients. However, the conclusions were contradictory.

This meta-analysis showed that the pooled value of Mean [95% CI] was of statistical significance, revealing increased circulating levels of betatrophin in T2DM. By subgroup analyses, (1) all the research samples for Chinese people were serum, and the results showed that betatrophin circulating level increased in the serum of Chinese T2DM patients; (2) betatrophin circulating level increased in the plasma of the T2MD patients. Since the study subjects are all Caucasian, we could make a conclusion for the moment: increased plasma betatrophin circulating levels in Caucasian T2DM patients.

However, the result showed that circulating levels of betatrophin in Caucasian T2DM patients have no statistical significance. There are differences between the result of this subgroup analyses and the overall result. Nevertheless, the results were consistent with the conclusions by Fenzl et al. [7]. In previous research [7], plasma betatrophin concentrations did not differ between patients with type 2 diabetes and nondiabetic controls. However, there were no sufficient theories to illustrate these results. So the exact mechanisms of betatrophin action in diabetic disease are still needed.

During the screening, Ebert et al. [20] also consider that betatrophin will increase in the serum of T2DM patients. However, the result of the article was presented by Median [interquartile range] and it cannot be converted into Mean ± SD, so we have to exclude it.

In this meta-analysis, the entire included case group is T2DM patients. Since the pathogenesis of T1DM is different from that of T2DM, we have not included articles on T1DM research. There have been scholars who have explored, among which Espes et. al. [21] studied betatrophin circulating level in the plasma of the Swedes. The study result showed that betatrophin circulating level in the serum of the T1DM patients is as twice as the normal glucose tolerance group (300 pg/mL). The article has also mentioned that betatrophin circulating level changes with different age groups in the healthy groups. More efforts are encouraged to explore this association.

According to the forest plot A, substantial heterogeneity (*I* ^2^ = 99%) was observed among the studies. To find the source of heterogeneity, sensitivity analysis was performed. Articles were excluded one by one before reanalyzing statistically. The results showed that there was always substantial heterogeneity. Heterogeneity increases were caused by selection bias existing in case group. The second was the small sample size. Different race and different sample are also the reasons of heterogeneity, because of which we conduct subgroup analyses. Fu et al.'s [22] research claims that different research results about the betatrophin circulating level in T2DM patients are caused by different ELISA research kits used and it may be also the reason of heterogeneity. A nonsignificant *P* value according to Begg's and Egger's tests (Begg, *P* = 0.11 Egger, *P* = 0.25).

This is meta-analysis investigating the association between betatrophin and DM, which has significantly increased the statistical power. However, the present results of meta-analysis have some limitations. First, DM is a kind of disease influenced by multiple factors and there are complex interactions between them. In this meta-analysis we have not enough data to evaluate the interaction of betatrophin in diabetes and other factors. Second, we have no access to the data that are not published, so the publication bias cannot be avoided absolutely. Third, our search languages are only English and Chinese and research data of other races may have influence on the results. Fourth, we did not get the original data of the included literature, so we cannot guarantee the accuracy of the data.

Despite the limitations above, we believe that based on the positive results of the meta-analysis, it is worth for more scholars making further study in prospective study and follow-up research.

In conclusion, the meta-analysis of all published case-control studies on betatrophin and T2DM revealed increased circulating levels of betatrophin in patients with type 2 diabetes.

## Figures and Tables

**Table 1:** Characteristics of eligible studies included in the meta-analysis.

First author	Publication year	Country	Sample	Methods	Cases		Controls	
					Subjects (*n*)	Betatrophin (Mean ± SD, pg/mL)	Subjects (*n*)	Betatrophin (Mean ± SD, pg/mL)
Fenzl [7]	2014	Germany	Plasma	ELISA	18	1646.5 ± 551.12	19	1643.4 ± 1002.11
Chen [8]	2014	China	Serum	ELISA	112	798.6 ± 449.78	137	692.7 ± 339.44
Espes [9]	2014	Sweden	Plasma	ELISA	27	893 ± 415.69	18	639 ± 280.01
Fu [10]	2014	USA	Serum	ELISA	14	5560 ± 2731.41	15	2190 ± 929.52
Hu [5]	2014	China	Serum	ELISA	83	613.08 ± 65.15	83	296.57 ± 52.16
Gómez-Ambrosi [6]	2014	Spain	Serum	ELISA	15	13500 ± 8800	33	45100 ± 24400
Wang [11]	2015	China	Serum	ELISA	35	1123.52 ± 319.01	31	669.15 ± 318.16
Xie [12]	2014	China	Serum	ELISA	50	412.53 ± 237.67	50	306.20 ± 283.82
Yamada [13]	2015	Japan	Serum	ELISA	30	1614 ± 647	12	300 ± 236
Guo [14]	2015	China	Serum	ELISA	56	162.56 ± 42.25	60	123.52 ± 28.19
Xie [15]	2015	China	Serum	ELISA	50	833.45 ± 456.21	50	541.68 ± 136.2

**Table 2:** All the forest plot data summary.

Subgroup	Article number	Total (95% CI)	*I* ^2^	*Z*	*P*	Model
Chinese	6	214.15 [57.65, 370.65]	99%	2.68	*P* = 0.007	Random
Caucasian	4	98.40 [−1585.08, 1781.88]	95%	0.11	*P* = 0.91	Random
Plasma	2	220.47 [31.27, 409.67]	0	2.28	*P* = 0.02	Fixed
Serum	9	358,64 [198.04, 519.24]	99%	4.38	*P* < 0.0001	Random
Total	11	329.46 [182.51, 476.42]	99%	4.39	*P* < 0.0001	Random
